# Enhancing shipyard transportation efficiency through dynamic scheduling using digital twin technology

**DOI:** 10.1371/journal.pone.0297069

**Published:** 2024-02-29

**Authors:** Miaomiao Sun, Chengji Liang, Daofang Chang

**Affiliations:** 1 Institute of Logistics Science and Engineering, Shanghai Maritime University, Shanghai, China; 2 Logistics Engineering College, Shanghai Maritime University, Shanghai, China; University of Victoria / Universiti Teknologi Malaysia /, CANADA

## Abstract

Uncertainties, such as road restrictions at shipyards and the irregular shape of blocks, pose challenges for transporter scheduling. Efficient scheduling of multiple transporters is critical to improving transportation efficiency. The digital twin (DT) technology offers numerous benefits, enabling interactions between the virtual and real worlds, real-time mapping, and dynamic performance evaluation. Based on DT technology, this study proposes a dynamic scheduling approach for cooperative transportation utilizing multiple transporters. The scheduling problem for multiple transporters is addressed and modeled in this study, considering factors such as block size and transporter loading. To solve this problem, a framework of DT-based multiple transporters system is established in a virtual environment. By inputting block information into this system, a solution is generated using transporter scheduling rules and interference detection methods. Experimental comparisons are conducted in this paper, exploring various scenarios with different number of tasks and the application of DT. The results demonstrate that the proposed approach effectively enhances transportation efficiency and improves ship construction efficiency. Hence, this study expands the application of DT technology in dynamic scheduling of transportation in shipyards and provides new ideas for shipbuilding company managers.

## 1 Introduction

Transportation is a crucial element in the global economy and has a significant impact on international trade and commerce. Sea transportation, in particular, a critical factor in facilitating the movement of goods and services across the globe. Now, about 90% of world trade is transported by the international shipping industry [1–5]. With the surge in demand for container shipping, global maritime trade rebounded in 2021. Freight volume is projected to grow by 3.2%, reaching 11 billion tonnes. This represents a 7-percentage-point increase compared to the 3.8% decline in 2020 [6]. Considering the significance of the shipbuilding industry, it becomes essential to address challenges related to optimizing production timelines and resource allocation in the shipbuilding process. One proposed solution is the innovative use of DT technology, which enhances transportation efficiency in shipyards. By adopting DT technology, shipbuilders can streamline operations, improve coordination, and make informed decisions, ultimately contributing to the improvement of shipbuilding efficiency and the overall development of the maritime industry.

The body of an entire ship comprises numerous units, known as blocks, which must require various operations before overall assembly. These blocks need to be combined according to strict process requirements, and several processes may need to be performed simultaneously. Timely and efficient transportation of these blocks is crucial to ensure the smooth implementation of subsequent production plans, as any delays can affect the entire shipbuilding cycle and ultimately lead to postponed ship loading and delivery times. The transportation of blocks at shipyards is carried out using specialized vehicles called transporters. For smaller block volumes, a single transporter can transport multiple blocks simultaneously. In cases with a larger number of blocks, the use of multiple transporters for cooperative transportation may be necessary. However, this process is fraught with uncertainties, such as road obstacles, narrow road widths, or other transportation constraints, which must be accounted for. Therefore, the effective reduction of block scheduling costs, improvement of multiple transporter scheduling efficiency, and the rational planning of block production scheduling processes are crucial for shortening the shipbuilding cycle, and enhancing shipbuilding efficiency.

Thanks to continuous advancement and implementation of information and communication technology in shipyards, the real-time online scheduling system has emerged to meet the demands of the times, allowing for precise positioning of transporters and ship blocks. The significance of this system lies in its ability to plan real-time transportation routes, thereby enhancing transportation efficiency, reducing delays, and optimizing overall ship production progress. DT, or DT technology, integrates multiple physical, scale, and disciplinary attributes, providing real-time synchronization, precise mapping, high-fidelity simulation, and integration of the physical and information worlds. It was originally introduced by Michael Grieves and John Vickers during the product lifecycle management program at the University of Michigan in 2003 [7]. Since then, DT has gained increasing prevalence across various industries. With the rapid development of information technologies such as IoT, artificial intelligence, and 5G networks, the applications of DT have expanded even further. Today, DT is utilized in a diverse range of industries, including port and logistics, healthcare, aerospace, and more. The smart manufacturing industry widely recognizes DT as a highly effective means of integrating the physical and information worlds.

DT is a physical product mirror that utilizes a multidisciplinary, multi-scale sim-elation process. It leverages physical models, sensors, and historical data to reflect the entire product lifecycle. Firstly, DT allows for the conversion of the physical model of the shipyard into an information model, enabling enhanced analysis and decision-making. Through automated data collection devices like RFID, sensors, and cameras, information is collected from various objects to reflect their current status. The use of DT technology facilitates monitoring and modelling of all production elements, including the transportation of multiple transporters between shipyard block yards. Additionally, establishing basic 3D modelling of elements such as yards, blocks, trans-porters, workers, drivers, containers, roads, etc., and defining rules for dynamic changes between block yards, such as block distribution and transporter truck routes, is essential. Second, multi-source heterogeneous data processing techniques are required to process, clean, filter, and integrate data from various sources. The synthesis enables the determination of real events between yards and drives the evolution of the inter-yard scheduling model. Finally, optimal control strategies are imposed based on the information model, enabling the evaluation of scheduling schemes and improving efficiency. This approach minimizes trial-and-error controls in the field and fosters tight interaction between the physical and information models.

In this context, we will be focusing on DT techniques. DT enables the creation of a virtual world where the real-time scheduling of transporters for block transportation between yards can be simulated and analyzed with clarity. The DT receives input data from the physical space, including the positions of blocks and the transportation status of multiple transporters between yards. Through feedback mechanisms, the virtual and physical spaces interact and iterate, aiming to achieve the optimal scheduling solution. This iterative process allows for dynamic adjustments and optimization in the physical space, resulting in improved scheduling efficiency and performance.

Among the typical vehicle scheduling problems, this study contributes to the lit-erasure on vehicle scheduling problems in several ways. Firstly, it presents a scheduling problem for cooperative transportation of multiple transporters dealing with irregularly shaped ship blocks and factors in the limited load-bearing capacity of transporters. Secondly, in comparison to previous literature, this study considers and simulates the dynamic uncertainties present in the scheduling environment, utilizing DTs for prediction and simulation. Finally, the GA is integrated within the virtual system built by the DT to optimize the scheduling and rescheduling scheme for transporters. The optimization encompasses factors such as transportation routes, loading/unloading sequences, and dynamic uncertainties.

The subsequent sections of this paper are structured in the following manner. Section 2 provides a comprehensive examination of the existing literature. Section 3 formulates a mathematical model that effectively eliminates non-value-added activities in transporter scheduling, aiming to optimize operational efficiency. Section 4 introduces a DT-based approach for proactive scheduling and improved coordination of multiple transporters. The implementation of a case study is explained in Section 5, highlighting its application and practical insights. Ultimately, Section 6 concludes the paper by summarizing the key findings and highlighting potential avenues for future research, such as improving scheduling efficiency, optimizing resource allocation, or addressing specific challenges in multiple transporters scheduling.

## 2 Literature review

The present investigation focuses on DT-based multiple transporters scheduling, with relevant literature classified into four distinct categories: transportation planning and truck scheduling, transporter scheduling, cooperative transportation and DT. To provide comprehensive picture of the most recent papers in each of these three areas, the subsections in this chapter will be organized around their respective studies. This organization will allow for a detailed examination of key findings, methodologies, and trends within each of the four categories.

### 2.1 Transportation planning and truck scheduling

Transport planning and truck scheduling are important research directions in the field of transportation. In recent years, researchers have proposed many new models and methods in these areas to optimize the efficiency of traffic networks and truck transportation, aiming to reduce travel time, minimize congestion, optimize resource allocation, and improve overall transportation system performance. The latest research has made significant contributions in areas such as route optimization, traffic flow management, demand forecasting, and intelligent transportation systems.

Firstly, in the aspect of traffic network optimization, researchers aim to minimize traffic congestion and reduce travel time by optimizing the topology and traffic flow of the network. The latest research utilizes various optimization techniques such as integer programming, linear programming, and graph theory to optimize specific components of the traffic network, including traffic signal timings, intersection design, and road network layout [8–10]. Secondly, in terms of path planning and route selection, researchers have developed efficient algorithms to find optimal driving paths and routes considering factors such as traffic conditions, road capacity, real-time data, and optimization criteria like fuel consumption or carbon emissions. These algorithms aim to minimize driving time, distance, and environmental impact. Furthermore, the latest research focuses on multimodal path planning, which involves integrating different modes of transportation, including walking, cycling, and public transportation, in route planning [11, 12]. For truck scheduling and delivery route optimization, researchers are committed to developing efficient truck scheduling algorithms to optimize truck itineraries and delivery routes, considering factors such as truck capacity, load restrictions, delivery windows, and transportation costs. These algorithms aim to minimize transport time, costs, and empty mileage. Recent research also explores truck sharing and consider multi-objective optimization approaches to improve logistics efficiency and reduce carbon emissions [10, 13, 14]. With the development of intelligent transportation systems, researchers are increasingly interested in real-time vehicle path tracking and dynamic scheduling. Utilizing technologies such as GPS, mobile internet, and sensor data to monitor truck positions and traffic conditions in real-time. Based on this information, real-time scheduling and path planning capabilities are enabled [15, 16].

In conclusion, the latest research in traffic planning and truck scheduling aims to improve traffic efficiency, reduce congestion, and optimize logistics efficiency. By developing efficient algorithms and models, the latest research significantly contributes to the sustainable development of urban transportation and logistics, promoting environmental sustainability and enhancing the overall quality of transportation and logistics systems.

### 2.2 Transporter scheduling

Transporter scheduling is a related research area in the field of truck scheduling and is also the research problem of this paper. Therefore, in this section, a comprehensive review of research in the field of transporter scheduling will be conducted, focusing on key methodologies, algorithms, and optimization techniques. The transporter transportation scheduling at shipyards addresses the simultaneous pickup and delivery and time windows. This problem aims to determines the optimal transportation time and means of transportation between pick-up and delivery locations while considering requirements such as capacity, priority, and time window constraints [17].

Many scholars have researched the problem of single transporter transportation at shipyards. Kim and Joo [18] assumed that the transportation demand of each block is predetermined in a static condition. Under concurrent machine scheduling conditions, considering factors such as order setting time and work process limitations, Park and Seo [19] studied the transporter scheduling and path problem at shipyards. Joo and Kim [20] proposed a method for determining the transit time and method of task blocks from the origin yard while considering delivery constraints. Liu et al. [21] considered a task block transportation storage problem, incorporating constraints related to block time windows and movement costs. Roh and Cha [22] focused on minimizing travel distance without loading transport transporters and avoiding interference between them. They developed a block transportation scheduling system using a hybrid optimization algorithm, which was applied to an actual block transportation scheduling problem in a shipyard. To accurately simulate the uncertainty and dynamics of block transportation, Chen and Tian [23] studied a method of multi-type block storage yards at shipyard. By projecting the status of ship blocks’ transfers and temporary storages, they capture the aforementioned uncertainty and dynamics. Finally, Kwon and Lee [24] solved the problem of spatial scheduling of large assembly blocks, with objectives such as minimizing makespan and achieving load balancing.

It is apparent that the researchers’ primary goal was to minimize overall completion time, expenses, or delays, with relatively less attention paid to considering the non-value-added time of the transportation personnel in their studies.

### 2.3 Cooperative transportation

So-called multiple transporters transport, in other words, it is a way to transport heavy or large blocks in cooperation with other transporters, and such transportation requires the cooperation of transporters. When two transporters cooperate to load the same cargo, they must maintain the same transport route and transport speed. Several researchers have studied different cooperative transport problems. In the problem formulation of aircraft, Ioachim et al. [25] introduced a novelty sort of constraints, related to synchronization with schedules, and an optimal solution is proposed. Li et al. [26] investigated a simultaneous scheduling problem with the goal of achieving accurate delivery with minimal cost in assembly and air freight for the consumer electronics supply chain. To coordinate the different activities in a supply chain solution, Zandieh and Molla-Alizadeh-Zavardehi [27] studied the synchronization between production and air transport scheduling with the help of a mathematical model. Salazar-Aguilar et al. [28] introduced an arc-shaped synchronous routing problem applicable to snow clearing operations. El Hachemi et al. [29] presented a synchronization problem between routing and scheduling, specifically applied to forestry and the same type as the log truck scheduling problem. In some cases, Reinhardt et al. [30] considered the transportation of passengers as a multimodal problem with simultaneous constraints. Salazar-Aguilar et al. [31] addressed a synchronous routing problem for arcs and nodes, inspired by practical applications in road marking operations. Lee et al. [32] developed a supply chain model that integrates supplier multi-buyer inventory with transportation synchronization. For the scheduling of transporters in assembly areas at shipyards, N.-R. Tao et al. [33] proposed a metaheuristic algorithm considering constraints such as task block order and transportation cooperation. Jiang et al. [17] created a heuristic optimization algorithm to tackle the challenge of scheduling eco-friendly transportation with multiple vehicles and a single cargo in shipyards.

However, few scholars have taken into account the dynamic uncertainty in the scheduling process when addressing cooperative transportation scheduling problems.

### 2.4 DT

Research referring to DT has also inspired numerous studies in recent years. Dynamic scheduling methods are crucial for modern manufacturing systems due to the unpredictable nature of events that can occur during production. Examples of unforeseen circumstances in a production system include task insertions, order cancellations, workers’ absenteeism, and machine and equipment breakdowns [34]. In response, researchers have proposed various approaches to improve scheduling accuracy and efficiency. For instance, Zhang et al. [35] presented a DT model with five dimensions for workshop settings and investigated the techniques for predicting machine availability, detecting interference, and evaluating performance using this model. To overcome the limitations of traditional scheduling methods, Villalonga et al. [36] proposed a new framework that utilizes DT aggregation. Zhang et al. [37] devised a two-tier distributed architecture for dynamic shop floor scheduling, integrating DT scheduling agents for shop floor and multiple service units. These innovative approaches demonstrate the potential of DT technology to enhance manufacturing scheduling processes. Neto et al. [38] have developed a decision support system that utilizes real-time opportunities, such as supply shortages, temporary machine idleness, and equipment breakdowns, to minimize overall production penalties while scheduling preventive maintenance interventions. Rao Pabolu and Shrivastava [39] proposed an optimal dynamic solution for job rotation of fatigued workers on assembly lines, utilizing machine learning-based DT. Frantzén et al. [40] developed a new approach to optimization and data analysis using genetic programming heuristics and simulations running on DT. In terms of process simulation and production scheduling in the food processing industry, Koulouris et al. [41] discuss the application of integrated process and DT models. H. Zhang et al. [42] presented a multi-layered modelling framework that spans from the unit level to the system level, addressing the challenge of implementing model building from different perspectives on temporal and spatial scales. Z. Liu et al. [43] developed an intelligent scheduling approach utilizing DTs and super networks to generate process plans quickly and efficiently. Zhao et al. [44] proposed a DT-based approach to multi-crane scheduling and crane quantity selection.

Based on the above studies, we can conclude that DT can be effectively used to predict the dynamic behavior and scheduling of multiple transportation vehicles, thereby better improving shipbuilding efficiency. With the use of DT technology, dynamic uncertainties in transporter movements can be predicted and evaluated in advance. As a result, we present a study on how the scheduling of multiple transporters can be improved, for example, by optimizing routes, reducing idle time, or minimizing conflicts, using this technology.

## 3 Problem formulation

Following the literature review in the previous section, this section focuses on the specific research problem and mathematical model of this paper. Therefore, this section will start with two subsections, "Problem description" and "Model formulation".

### 3.1 Problem description

There are many sections between ship blocks, and each section has to go through multiple processes such as processing and cutting. These processes need to be completed separately in different yards. Therefore, transporters play a crucial role in shipyards scheduling. In the actual transportation process of blocks, due to other uncertain constraints such as irregular length, width and height, large volume and weight, and the height of the road, it is likely that the transporters cannot pass through the obstructed road section or affect its transportation scheduling efficiency. This represents a typical multi-vehicle-one-cargo dynamic scheduling problem. The problem of multiple transporters of block transportation scheduling at shipyards can be described as follows.

There exist a variety of transportation tasks (indexed by *i*,*j*∈{1,2,…,*n*},*i* ≠ *j*) based on production requirements; The transportation of blocks within shipyards, from the starting yard to the destination yard, is performed using special vehicles called transporters, which are indexed by *t* ∈*T* = {1,2,…,*t*}. Various types of transporters generally have a capacity ranging from 200-300 tons, with some capable of carrying up to 500 tons. Block transportation primarily relies on heavy-duty transporters for movements between the work yard and storage yard. However, there are limited resources of transporters available within the shipyards. Currently, the maximum load-bearing capacity of an existing shipyard transporter is 450 tons. When a single block exceeds this capacity, it is referred to as a large block and requires multiple transporters transportation.

**Fig 1:**
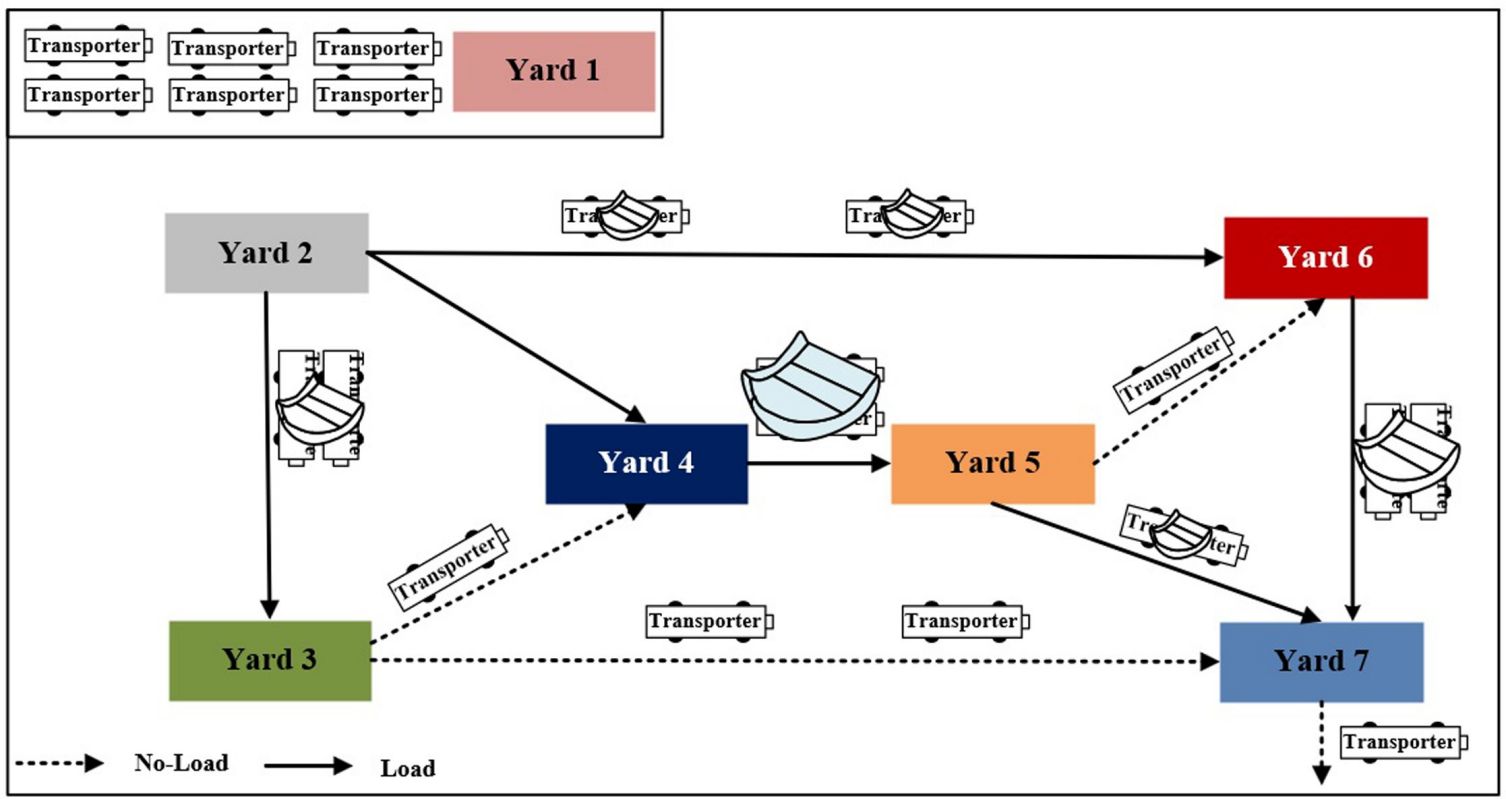
The principle of multiple transporters transportation.

The principle of multiple transporters transportation in the block yard is presented in Fig 1. The transportation includes information such as task, block ID, block weight capacity, starting yard code, destination yard code, and time window. The flatbeds are characterized by their car number and load capacity.

The underlying assumptions for this question are as follows.

1. The transporters cannot be aborted during a block transporter mission.
2. The weight of the large blocks must not exceed the combined load capacity of the two transporters.
3. Both transporters must maintain the same speed during cooperative transport.
4. Each block has a time window and all blocks must be released before they can be transported.
5. Block beyond the release time or expected time is subject to penalty.
6. Task blocks and constraint blocks are in the task sequence of the transporters and their sequencing needs to be satisfied when scheduling.
7. The extraction and stacking of blocks are an important part of segmented transport and cannot be ignored.
8. Transporters maintain a consistent speed regardless of their load status.
9. The analysis focuses on road access conditions within the block yard and does not consider external road conditions.

The assumptions in this paper are mainly based on two articles, such as [29, 33]. Based on the above assumptions, this paper will address the sequence of transport tasks and the best transport routes for the transporters, with the starting execution time of every block.

### 3.2 Model formulation

The sets, parameters and decision variables associated with the model are defined in Table 1.

**Table 1:** Notation.

Notation		Meaning
Sets	*B*	block number set {1,2,…,*n*},*i*,*j* ∈*B*, where *i* ≠ *j*
	*T*	Transporters number set *T* = {1,2,…,*t*},*t*∈*T*
	*S*	Transporters speed set *S* = {3,6}, *lsp* means load speed is 3*m*/*s*, means no-load speed is 6*m*/*s*
	*M*	Any large positive number
	*H*_*i*_	When *H*_*i*_ = 1represents the block *i* is large; conversely *H*_*i*_ = 0 represents the block *i* is small, that is to say, a transporter to travel it
Parameters	[*e*_*i*_,*l*_*i*_]	Task time window; *e*_*i*_ is the earliest start transporting time for task *i*;*l*_*i*_ is the latest start transporting time for task *i*
	[*E*,*L*]	Parking area time window parking area; *E* is the opening time; *L* is the closing time
	*w*_*i*_	Load capacity of task *i*
	*cw*_*t*_	Load capacity of transporter *t*
	*α*	Weighting factor of unloaded transport time for transporter
	*β*	Weighting factor of waiting time for transporter
	*dis*_*i*_	Distance between starting yard and destination yard of task *i*
	*dis*_*ij*_	Distance travelled by a flatbed truck from the end yard of task *i* to the start yard of task *j*
	*y*_*it*_	The 0-1 decision variable of final task *i* for transporter *t*
	$b_{i}^{s}$	The actual start time of task *i*
	$b_{i}^{e}$	The execution time of task *i*
Decision variables	$x_{0i}^{t}$	The 0-1 decision variable for transporter t to perform the first task *i*
	$x_{i0}^{t}$	The 0-1 decision variable for transporter t to perform the last task *i*
	$t_{ij}^{u}$	Starting point of task after performing task *i*, when *i* = 0 or *j* = 0, it means that the transporter goes to the parking lot
	$x_{ij}^{t}$	The decision variable of the order to perform for transporter *t*,when *i* = 0 or *j* = 0, it means that the transporter goes to the parking lot

The scheduling problem at hand can be formulated as a mixed integer linear problem with the objectives:

$$\begin{array}{c} MinU=\alpha \cdot \left(\sum_{t\in T}\sum_{j\in B\setminus i}\sum_{i\in B}x_{ij}^{t}\cdot t_{ij}^{u}+\sum_{t\in T}\;\sum_{i\in B}\;x_{0i}^{t}\cdot t_{0i}^{u}+\sum_{t\in T}\;\sum_{i\in B}\;x_{i0}^{t}\cdot t_{i0}^{u}\right)\\ +\beta \cdot \sum_{t\in T}\;\sum_{j\in B\setminus i}\;\sum_{i\in B}\;x_{ij}^{t}\cdot \left(b_{j}^{s}-b_{i}^{s}-b_{i}^{e}-t_{ij}^{u}\right) \end{array}$$ (1)

Objective (1) aims to minimize the total transportation time for the transporters, excluding any non-value-added time. The time consists of three parts: the unloaded transport time of one task block and another neighboring task block by the transporter, and the transporter the unloaded transport time including departure and back to parking area, and the not working time of the transporters ahead of time window. In this paper, the weighting coefficients *α* and *β* mentioned here represent the balance between the empty running time and waiting time of the transporter when performing tasks. Considering the actual situation of the shipyard, there may be differences in the no-load time and waiting time of the transporter during different stages of the transportation process, and therefore they need to be adjusted through weighting coefficients. In this study, we determined that both *α* and *β* are 1 by references [17, 18, 20, 22] and repeated experiments.

The constraints of this paper can be categorized as assignment constraints, which include constraints (2) to (9).


$$\sum_{i\in B}y_{it}=1+H_{i},\forall t\in T$$ (2)

$$e_{i}\leq t_{ij}^{u}\leq l_{i},\forall i,j\in B,i\neq j$$ (3)

$$b_{i}^{s}+b_{i}^{e}+t_{i0}^{u}\leq L,\forall i\in B$$ (4)

$$E\leq b_{i}^{s}-t_{0i}^{u},\forall i\in B$$ (5)

$$w_{i}\leq \sum_{t\in T}y_{it}\cdot cw_{t},\forall i\in B,t\in T$$ (6)

$$b_{i}^{s}+b_{i}^{e}+t_{i0}^{u}\cdot x_{ij}^{t}-b_{j}^{s}\leq \left(1-H_{i}\right)\cdot M,\forall i,j\in B,i\neq j,t\in T$$ (7)

$$\sum_{j\in B\setminus i}x_{ji}^{t}+x_{0i}^{t}=y_{it},\forall i\in B,t\in T$$ (8)

$$\sum_{j\in B\setminus i}x_{ij}^{t}+x_{i0}^{t}=y_{it},\forall i\in B,t\in T$$ (9)

The constraint (2) guarantees that the normal block is only one transporter required for transportation, and then the large block requires two transporters synchronization transportation. The constraint (3) indicates that the time window of block. The constraint (4) is the close time of the park area. The constraint (5) represents that the start time of the park area. The weight of block must not exceed the load capacity of the transporters which is represented by constraint (6). The constraint (7) states that the large block can only be executed when both transporters have arrived at the starting yard. In constraint (8), each task block is assigned to only one transporter, ensuring that it appears once. Constraint (9) enforces the same condition for all task blocks.

The assignment constraints are constraint (10) to constraint (14).


$$\sum_{i\in T}\;x_{0i}^{t}=1,\forall t\in T$$ (10)

$$\sum_{i\in t}\;x_{i0}^{t}=1,\forall t\in T$$ (11)

$$b_{i}^{s}+b_{i}^{e}+x_{ij}^{t}\cdot t_{ij}^{u}-b_{j}^{s}\leq \left(1-x_{ij}^{t}\right)\cdot M,\forall i,j\in B,i\neq j,t\in T$$ (12)

$$x_{ji}^{t}+x_{ij}^{t}\leq 1,\forall i,j\in B,i\in j,t\in T$$ (13)

$$\sum_{j=1,j\neq i}\;x_{ij}^{t}\leq y_{it},\forall i,j\in B,t\in T$$ (14)

The constraint (10) shows that each transporter only has a first task. The constraint (11) shows that each transporter just has a final task. Constraint (12) defines the start time of the next block based on the upper limit of the previous task block’s end time ($x_{ij}^{t}$) for the corresponding transporter. Constraint (13) prohibits the repetition of consecutive task blocks. Constraint (14) defines the feasible range of values for the decision variables, taking into account the specific constraints and requirements of the problem.

## 4 DT based scheduling approach

As mentioned earlier, transporters scheduling is difficult due to the dynamic behavior of the transportation system. To enable pre-analysis of various scheduling scenarios and simulate the behavior, a virtual transport system was utilized and integrated with the scheduling system. Thus, a new DT-based approach for multiple transporters scheduling was implemented. First, this section presents a DT-based optimization framework for scheduling multiple transporters in a shipyard block yard. Then, based on the framework, the method of transporter scheduling optimization is investigated. Next, the subsections "DT framework" and "Approach for scheduling transporters based on GA" are discussed.

### 4.1 DT framework

**Fig 2:**
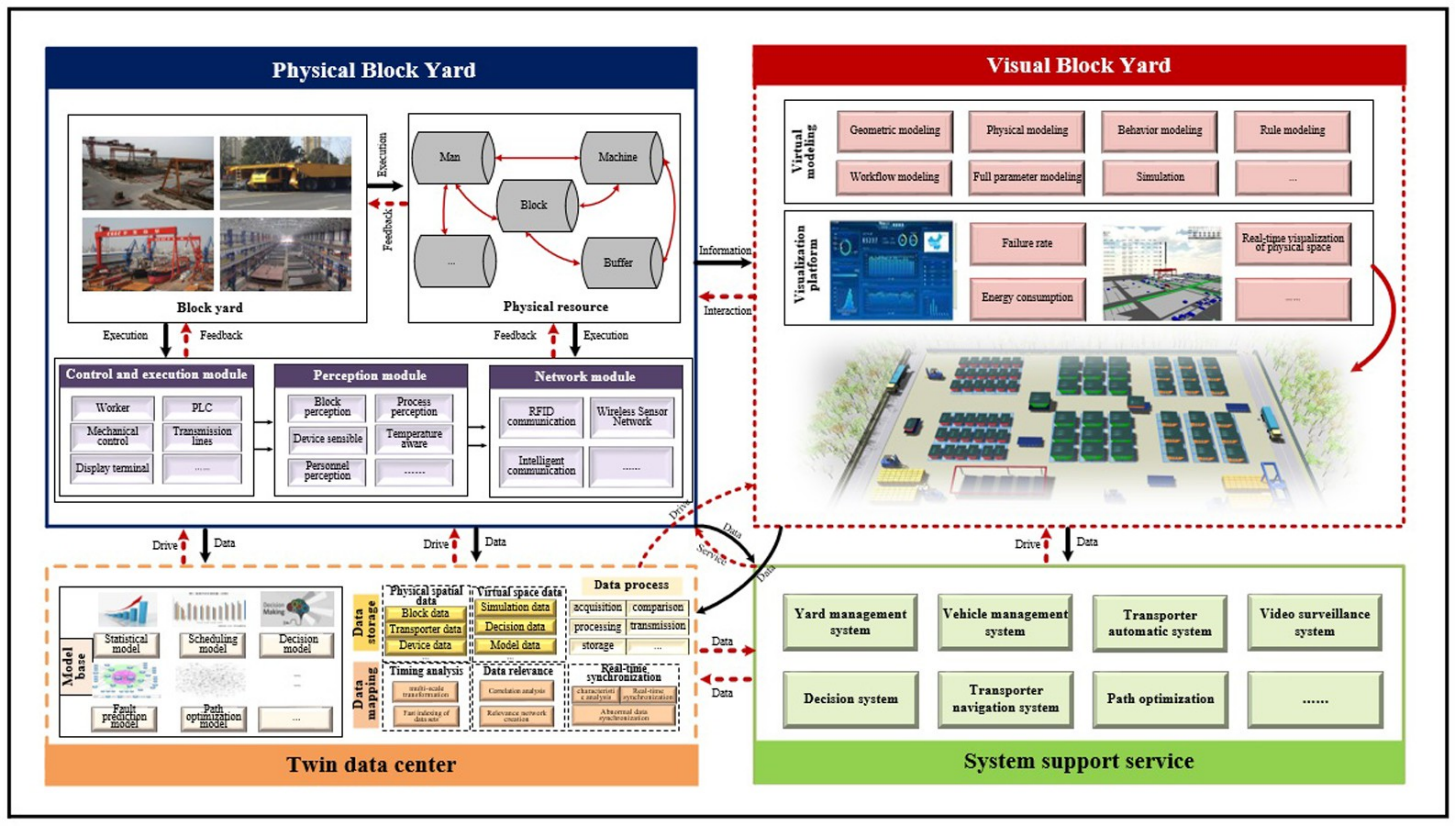
DT-based architecture of block transportation.

Building upon the work of Tao et al. [45] in DT-driven product design, manufacturing, and service, our study has developed a DT-based framework for transporter scheduling that addresses dynamic uncertainty, as illustrated in Fig 2. The framework consists of five essential components: a physical block yard, a virtual block yard, a twin data center, system support services, and the connections and interactions among these elements.

Based on the above framework and model, the DT-based framework for multiple transporters of block transportation scheduling optimization at shipyards are fully utilized. The objective is to achieve dynamic scheduling of multiple transporters by integrating real-time data and historical data generated from cooperative blocks transportations. Additionally, the framework drives relevant services within the system through a data service platform to effectively manage uncertainties in the scheduling process of transporters.

**Fig 3:**
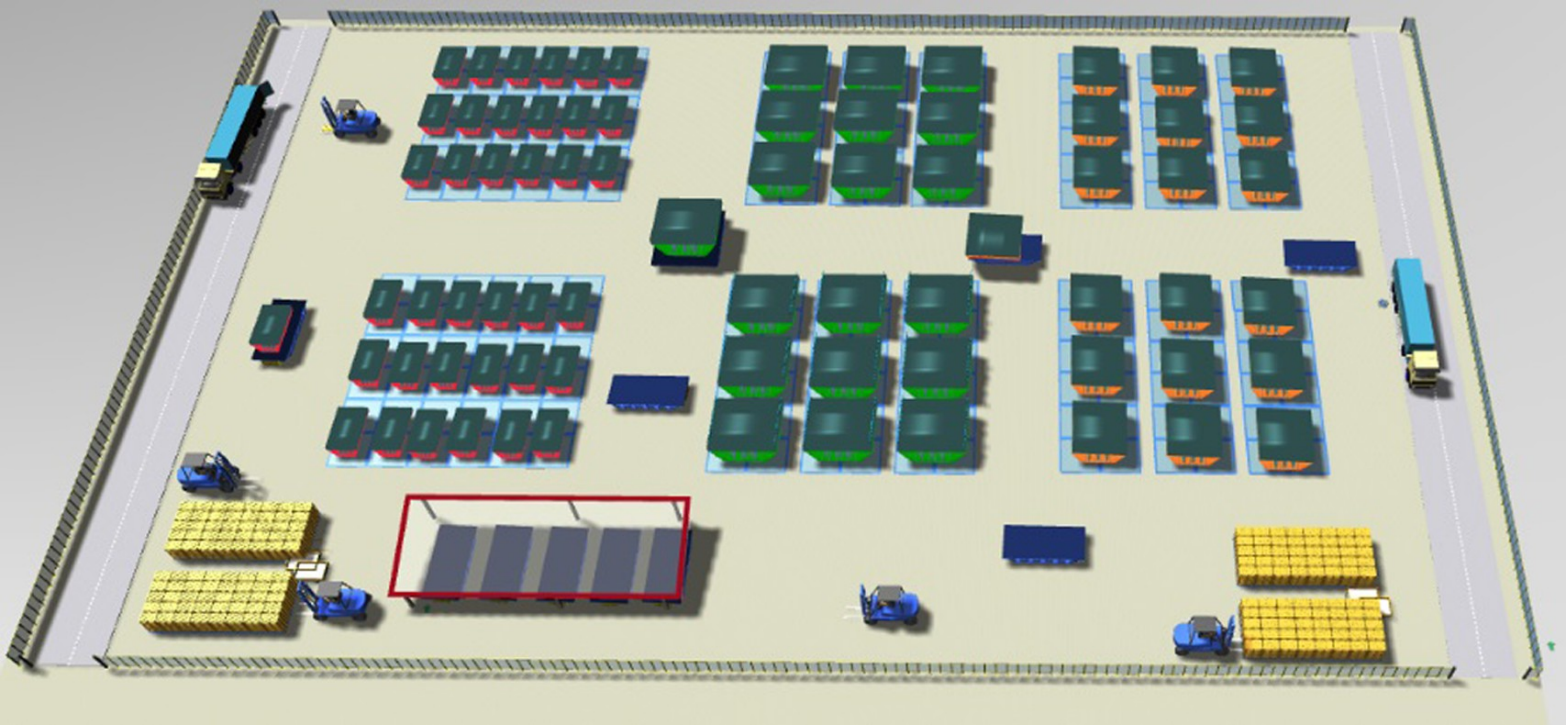
The process of a DT-based multiple transporters scheduling.

To facilitate the comparison, we utilize Unity and Plant-simulation to construct a 3D twin space, accurately replicating the real scene on a 1:1 scale, as shown in Fig 3. We construct 3D DT models of task blocks, yards, and transporters to create a comprehensive virtual model that incorporates actual transportation data from the shipyard. This ensures the verification of virtual-real consistency.

### 4.2 Approach for scheduling transporters based on GA

While minimizing redundant trips by transporters can assist in uncertainty management, modifying task sequences and assignments may introduce trade-offs between efficiency and potential contingencies. In the case of *t* transporters and *i* tasks, there can exist a large number of possible scenarios *t*^*i*^, making multiple transporters scheduling a challenging NP-hard problem. To address this challenge, various researchers have employed different methods, such as branch-and-check (Diefenbach et al. [46]; Li and Zhou [47]) and heuristic methods (Yin et al. [48]; Sistig and Sauer [49]). Among these methods, GA has demonstrated good performance and robustness in scheduling problems (Xu et al. [50]; Wu and Pang [51]; He et al. [52]). Therefore, GA was employed as the transporter scheduling method in this study.

**Fig 4:**
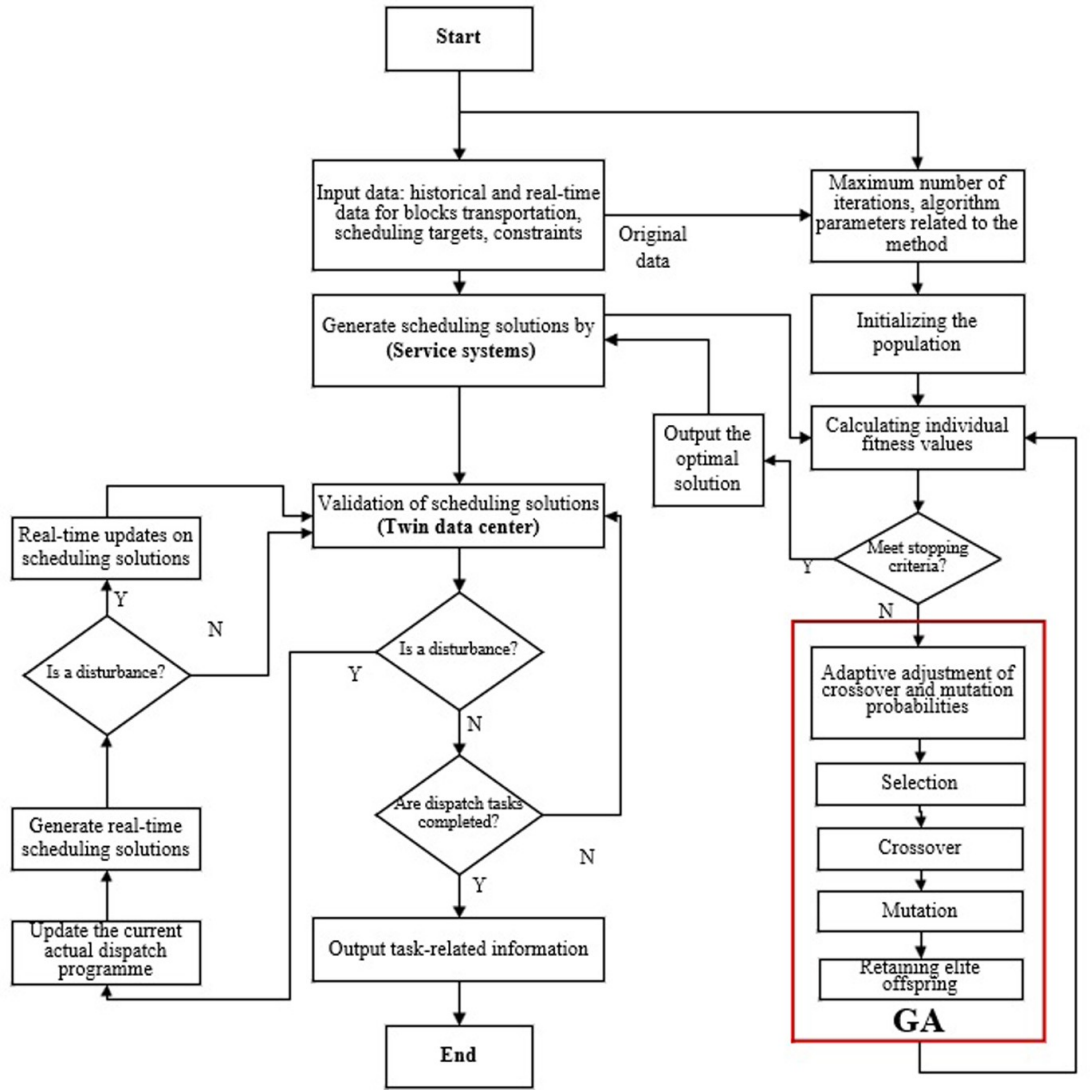
The process of a DT-based multiple transporters scheduling.

The flowchart illustrating the integration of GA and the simulation process is presented in Fig 4.

#### 4.2.1 Chromosome representation

**Fig 5:**
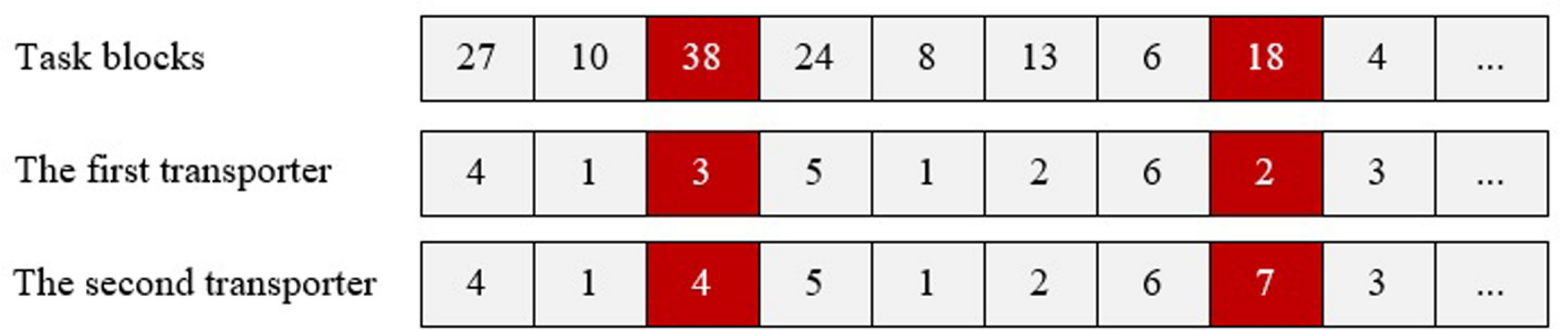
Three-dimensional coding and the associated initial feasible solution.

The first step is to define the encoding scheme. A critical aspect in GA design is the representation of chromosomes, which plays a vital role in achieving effective solutions. In this study, a three-dimensional coding scheme is utilized to represent the chromosomes, enabling the generation of an initial feasible solution, as depicted in Fig 5. Fig 5 displays the chromosome representation, where the first row corresponds to block numbers (40 blocks in total). The second and third rows indicate the number of transporters(six transporters in total), facilitating the encoding scheme. Number of transporters is listed in the second and third rows of the chromosome, with a total of six transporter.

#### 4.2.2 Generating initial population

Initially, the order of task block is randomly generated. The transporters are then assigned to each task block based on the load capacity of each transporter. For large task blocks that exceed the capacity of a single transporter, two transporters are randomly selected while ensuring the weight capacity conditions are met. The initial settings include defining the transport speeds for unloaded and loaded transporters. During the calculation of the objective function, individuals that fail to meet the specified time window requirements are penalized. Throughout the continuous iteration, feasible options are gradually replaced with improved solutions.

#### 4.2.3 Calculation of fitness and selection of parents

To optimize the transportation process and reduce non-value-added travel time, the fitness function in the GA is designed based on the inverse of time. A higher fitness score is assigned to solutions that result in shorter travel times, aligning with the objective of minimizing non-value-added travel time. After the completion of a simulation run, the optimal solution is determined by selecting the solution with the lowest total time and its corresponding GA fitness score.

This paper uses elite selection strategy and roulette wheel selection method in the proposed approach. A set of chromosomes, referred to as parent chromosomes, is selected from the initial population using the roulette wheel selection method. These parent chromosomes contribute their genes to the offspring chromosomes in the next generation. Based on the current population, the best of each generation will form the next generation of s populations through an elite selection strategy. Generally, the selection process favors chromosomes with higher fitness values to become parents, ensuring that genetic material from solutions with better performance is passed on to the next generation.

#### 4.2.4 Crossover and mutation operation

**Fig 6:**
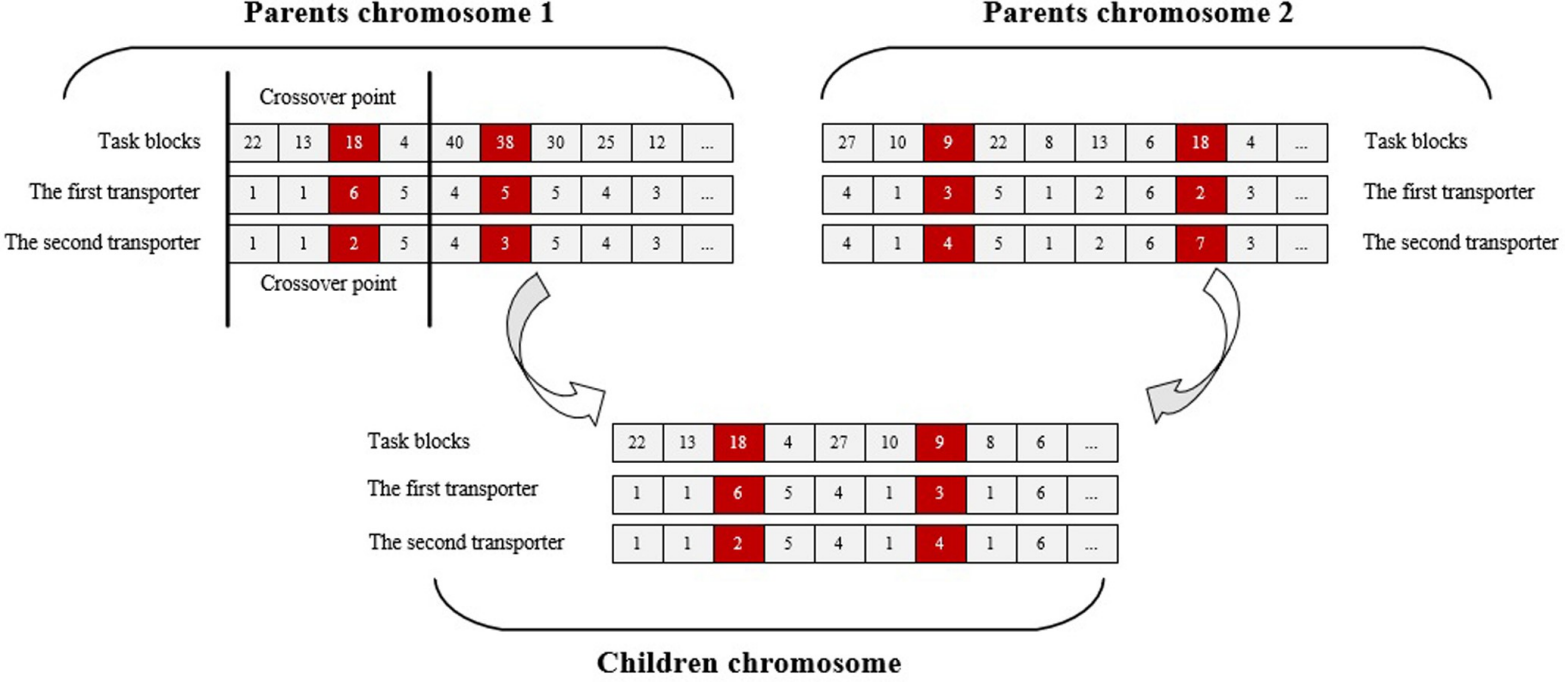
Example of single-point crossover operations.

Offspring are generated using a single-point mutation method, which involves altering a single gene within the chromosome. A crossover operation example is displayed in Fig 6. Once the selection operation is complete, parent individuals randomly to produce offspring. The crossover probability, which determines the likelihood of genetic material exchange between parental chromosomes, is determined by their crossover rate.

**Fig 7:**
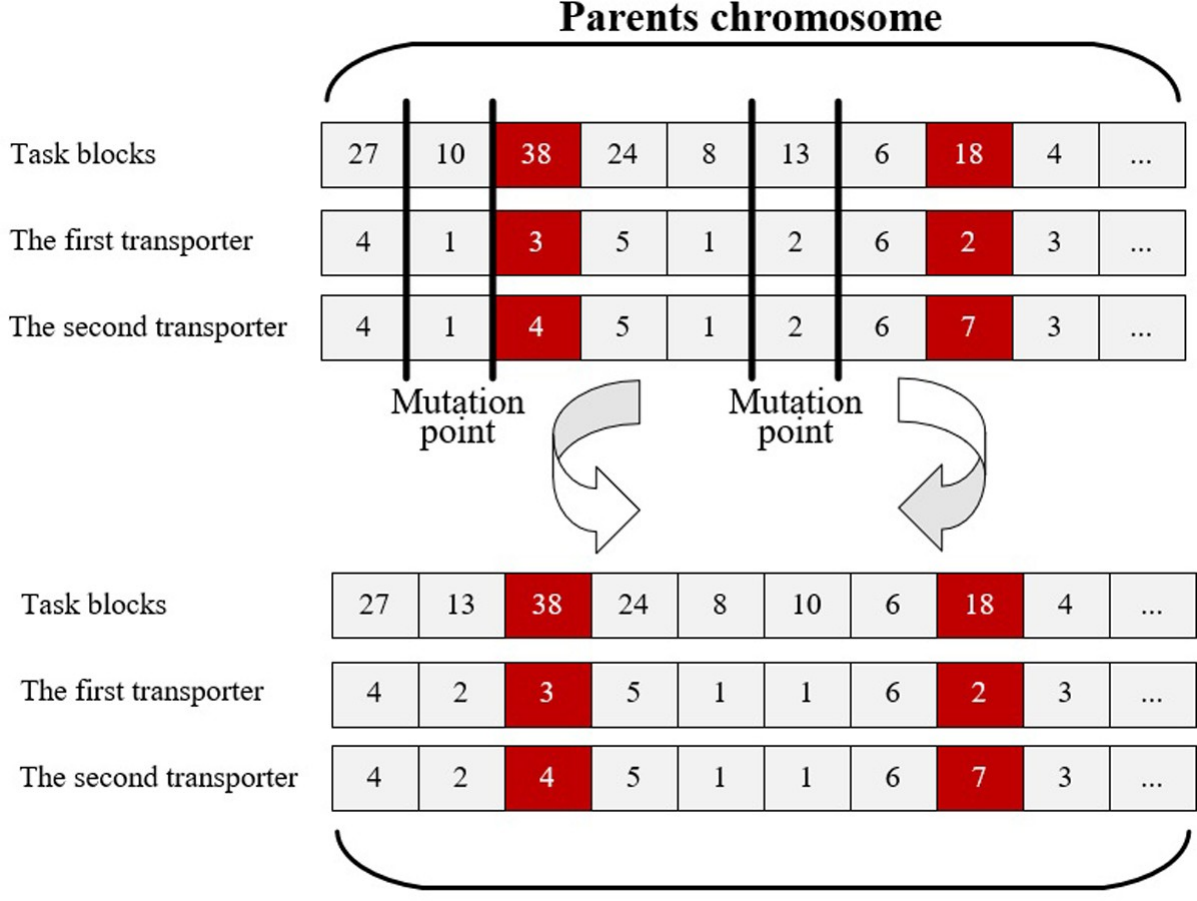
Example of swap mutation operations.

Chromosome mutation in GA serves a distinct purpose from crossover operations, introducing diversity and exploring new solutions. While sharing some similarities with crossover, chromosome mutation plays a separate role in the optimization process. The exchange mutation method involves two steps, as shown in Fig 7. Firstly, generating an index that corresponds to a specific block within the parent chromosome. And secondly, obtaining the new generation chromosome by swapping the indexed block with another block based on its chromosome value.

## 5 Computational experiments

To validate the effectiveness of proposed model and algorithm, this paper conducts experiments under various situations and scales to verify their performance. To enhance the practicality of the research, the generated data is derived from the actual scheduling data of the Shanghai Hudong-Zhonghua Shipyard, in terms of task block information, incorporating relevant information such as task block details, transporters information, and other relevant factors.

### 5.1 Case study and data generation

In this study, we assume that there are task blocks and transporters. In addition, we obtained detailed data from a shipyard for 40 groups of block tasks, including specific information such as starting yard, time window and load bearing, respectively, as shown in Table 2. In this paper, we refer to the blocks whose weight exceeds the load-bearing capacity of a single transporter truck as large block, e.g., the 9th block, the 18th block, the 28th block, and the 38th block in Table 2 are all large blocks. 

**Table 2:** Notation.

Task Blocks	Start Yard	Destination Yard	*e*_*i*_	*l*_*i*_	*w*_*i*_
1	2	4	0	200	238
2	5	6	0	200	244
3	6	3	0	200	264
4	5	7	0	200	276
5	4	5	0	200	280
6	3	4	0	200	283
7	2	4	0	200	283
8	6	3	0	200	288
9	7	4	0	200	450
10	5	3	0	200	220
11	6	7	50	250	220
12	5	3	50	250	222
13	4	6	50	250	226
14	3	5	50	250	226
15	2	3	50	250	242
16	6	5	100	300	258
17	2	4	100	300	276
18	6	3	100	300	450
19	7	2	100	300	319
20	5	6	100	300	326
21	3	7	150	300	238
22	5	3	150	300	244
23	4	7	150	300	264
24	5	3	150	300	276
25	4	7	150	300	280
26	3	6	150	300	283
27	2	4	150	300	283
28	6	3	250	520	450
29	2	3	250	520	200
30	6	5	250	520	220
31	5	3	250	520	220
32	4	6	250	520	222
33	3	5	250	520	226
34	2	3	250	520	226
35	6	5	250	520	242
36	2	4	350	520	258
37	6	3	350	520	276
38	7	2	350	520	450
39	5	6	350	520	319
40	5	7	350	520	326

Regarding the transporters, the number of transporters and their carrying capacity are shown in Table 3. And when the transporters are traveling unloaded, the speed is 3 m/s; when the transporters are loaded, the speed is 6 m/s.

**Table 3:** The data information for transporters.

Transporters Number	*cw*_*t*_
**1**	250
**2**	270
**3**	325
**4**	380
**5**	380
**6**	425

In addition to this, we have specified the distances between the starting yards in Table 1, which is shown in Table 4. It is worth noting that these experiments were conducted on advanced computers equipped with an Intel Core i5 processor, 4GB memory, MATLAB software, and CPLEX software, ensuring the highest level of accuracy and reliability.

**Table 4:** The distance information between yards.

Yard/distance	1	2	3	4	5	6	7
**1**	0	1600	400	600	1000	700	900
**2**	1600	0	1200	1000	600	1500	1300
**3**	400	1200	0	200	600	300	500
**4**	600	1000	200	0	400	500	300
**5**	1000	600	600	400	0	900	700
**6**	700	1500	300	500	900	0	200
**7**	900	1300	500	300	700	200	0

### 5.2 Parameter tuning

After completing the data set preparation in the preceding section, this study aims to conduct experimental investigations and research on parameter tuning in the current section to improve the effectiveness of metaheuristic algorithms in solving complex optimization problems. To achieve this goal, we utilize the Taguchi experimental method to fine-tune algorithmic parameters and reduce the number of iterations required. It is worth noting that this methodology has been employed in numerous prior investigations, as demonstrated by [53, 54]. Within this framework, the signal-to-noise ratio (S/N) is leveraged to optimize the response variable [55, 56], which is particularly suitable for mathematical models that aim to minimize the objective function (OF), as indicated by the following formula:

$$S/N=-10\times \log_{10}{\left(OF\right)}^{2}$$ (15)

In this study, the GA algorithm involves the adjustments of three parameters, including population size(*nPop*), crossover rate(*Pc*), and mutation rate(*Pm*). Similarly, the SA algorithm also has three parameters that need to be adjusted, including the number of sub-iterations (*N*), initial temperature (*t*_0_), and temperature reduction rate (*T*). Based on the literature of [53, 54], we have selected three potential values for each parameter, as shown in Table 5.

**Table 5:** Candidate values of the parameters of our algorithms.

Algorithm	Parameters	Candidate value 1	Candidate value 2	Candidate value 3
GA	*nPop*	200	300	500
	*Pc*	0.9	0.6	0.8
	*Pm*	0.1	0.05	0.2
SA	*N*	100	500	1000
	*t*_0_	10000	15000	20000
	*T*	0.1	0.01	0.001

For both algorithms, the Taguchi method determined that the optimal choice was an L9 orthogonal array, requiring only 9 experiments for each test problem. After running each algorithm 9 times for each test problem, we used relative percent deviation (RPD) from the Taguchi method to convert the optimal value of the response metric to a normalized value. RPD is defined as follows:

$$RPD=\frac{A{\text{lg}}_{sol}-Min_{sol}}{Min_{sol}}$$ (16)

In this study, we used *Min*_*sol*_ to represent the optimal solution and *A*lg_*sol*_ to represent the output result of the algorithm. It is worth noting that a smaller value of this metric indicates better performance. After defining the RPD for each test problem and computing the average RPD for all test problems, we determined the optimal values for each algorithm, which are presented in Table 6 based on the average RPD graph for each parameter.

**Table 6:** Tuned values of parameters of each algorithm.

Algorithm	Parameters
GA	*nPop* = 200,*Pc* = 0.9,*Pm* = 0.1
SA	*N* = 1000,*t*_0_ = 20000,*T* = 0.01

### 5.3 Comparisons with different task sizes

To facilitate a comparison between the two approaches, we integrate distinct task blocks using various numbers (*B* = {20,30,40,50,60}) and numbers of transporters (*T* = {4,5,6,}). Essentially, the GA is utilized to tackle the model, and subsequently, the outcomes are cross-checked with the exact solution obtained through the CPLEX software to verify the effectiveness of the proposed approach in this study.

We will compare the optimal values solved by the GA with the software-accurate values of CPLEX in the following points: the optimization model, min objective values and the transportation efficiency of transporters, penalty value and CPU time are calculated. Furthermore, the transportation efficiency is calculated as the ratio of the transporters’ load transport time to the total transport time, expressed as γ. The penalty value resulting from delays and number of transporters, represented as a proportion of the objective function value, is denoted by δ.

Table 7 presents the solution results and computational performance solved by the GA and obtained using the CPLEX software. From the above table we can see that the solution time for CPLEX software increases sharply, the more the number of tasks and transports increases. In the actual solution process, when the number of tasks is less than or equal to 20, CPLEX software will quickly find the optimal exact solution. But when the number exceeds 20, the GA algorithm outperforms CPLEX, verifying that the GA algorithm has good results in practical large-scale applications.

**Table 7:** Results about different task blocks and transporters between CPLEX and GA.

*B*	*T*	CPLEX			Mean CPU time	GA			Mean CPU time	
		*U*	*γ*	*δ*	*t*	*U*	*γ*	*δ*	*t*(*s*)	*GAP*
**20**	**4**	648.2	0.73	0.80	33.762s	648.2	0.72	0.81	136.854	0.00
**30**	**4**	912.6	0.64	0.83	>4h	844.2	0.66	0.80	177.658	-0.08
**40**	**4**	2013.5	0.65	0.95	>4h	1852.4	0.67	0.93	284.573	-0.09
**50**	**4**	2245.1	0.63	0.96	>4h	1651.1	0.63	0.95	362.754	-0.35
**60**	**4**	4193.4	0.64	0.98	>4h	3443.7	0.64	0.98	418.763	-0.21
**20**	**5**	514.9	0.73	0.67	20.295s	514.9	0.74	0.66	97.437	0.00
**30**	**5**	842.3	0.64	0.72	>4h	842.3	0.65	0.62	167.478	0.00
**40**	**5**	1843.7	0.63	0.63	>4h	1551.8	0.66	0.60	273.872	-0.18
**50**	**5**	2165.3	0.62	0.62	>4h	1726.7	0.63	0.61	346.482	-0.25
**60**	**5**	3886.1	0.64	0.64	>4h	2783.6	0.66	0.57	387.149	-0.39
**20**	**6**	369.5	0.69	0.45	15.047s	369.5	0.69	0.49	98.273	0.00
**30**	**6**	743.7	0.64	0.61	>4h	743.7	0.74	0.51	173.851	0.00
**40**	**6**	1699.3	0.61	0.83	>4h	1432.2	0.71	0.73	250.629	-0.18
**50**	**6**	2498.2	0.62	0.87	>4h	1887.4	0.63	0.77	319.802	-0.32
**60**	**6**	3359.6	0.59	0.92	>4h	2342.6	0.61	0.89	355.762	-0.43

GA has limitations in some sense that there is no guarantee that an optimal solution will be found. We can conclude from Table 7 that when the number of task segments is small, the difference between the exact solution and the optimal solution obtained by CPLEX is small, which can be allowed. At the same time, the GA can also quickly obtain better optimal solutions in large-scale quantities, which verifies the effectiveness of the method proposed by this paper in practical applications.

### 5.4 Comparisons with simulated annealing algorithms

Drawing on the numerical experimental data that we have prepared and comparisons that we have made using different task scale methods, our focus is to evaluate the GA that we have proposed in contrast to the simulated annealing algorithm, and subsequently, analyzing and comparing the results. Convergence analysis and result comparison will be carried out to achieve this objective. We have discussed the details of parameter tuning in subsection 4.2.

**Fig 8:**
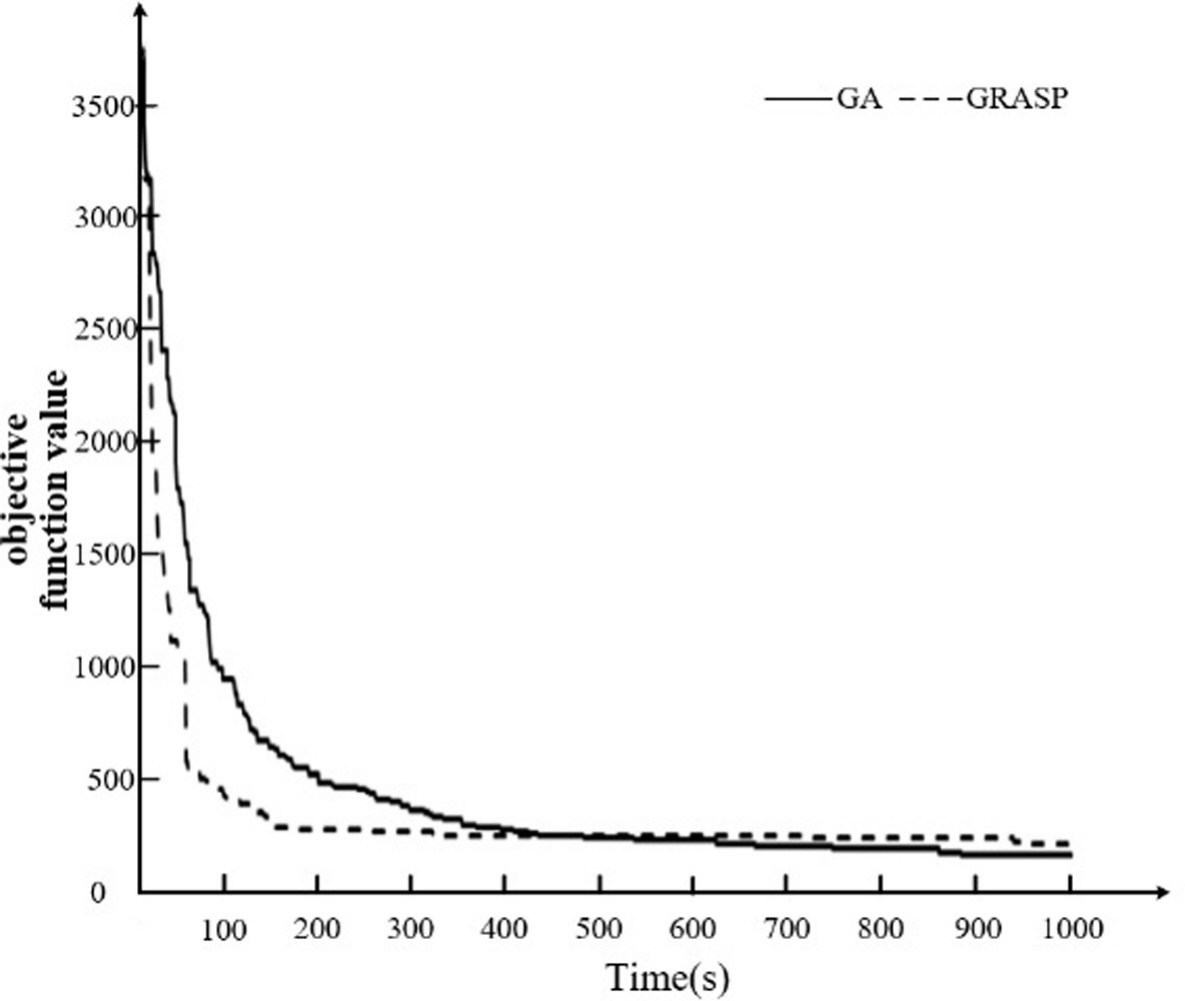
Comparison of algorithm convergence process.

To better compare the effectiveness of the GA, this section initially compares the convergence speeds of this algorithm and the simulated annealing algorithm from the convergence’s viewpoint, by iterating both algorithms for 1000 times. The GA has a slower convergence speed compared to the simulated annealing algorithm, however, the results obtained using the GA are better. Kindly find the details in Fig 8.

After analyzing the convergence of the two algorithms as discussed earlier, we now compare their scheduling results using the same iteration count of 1000 times while incrementing the number of block tasks from 20, 30, 40, 50, to 60, respectively. The results are then compared in terms of total non-value-added time, waiting time, no-load time, and postponement time.

**Fig 9:**
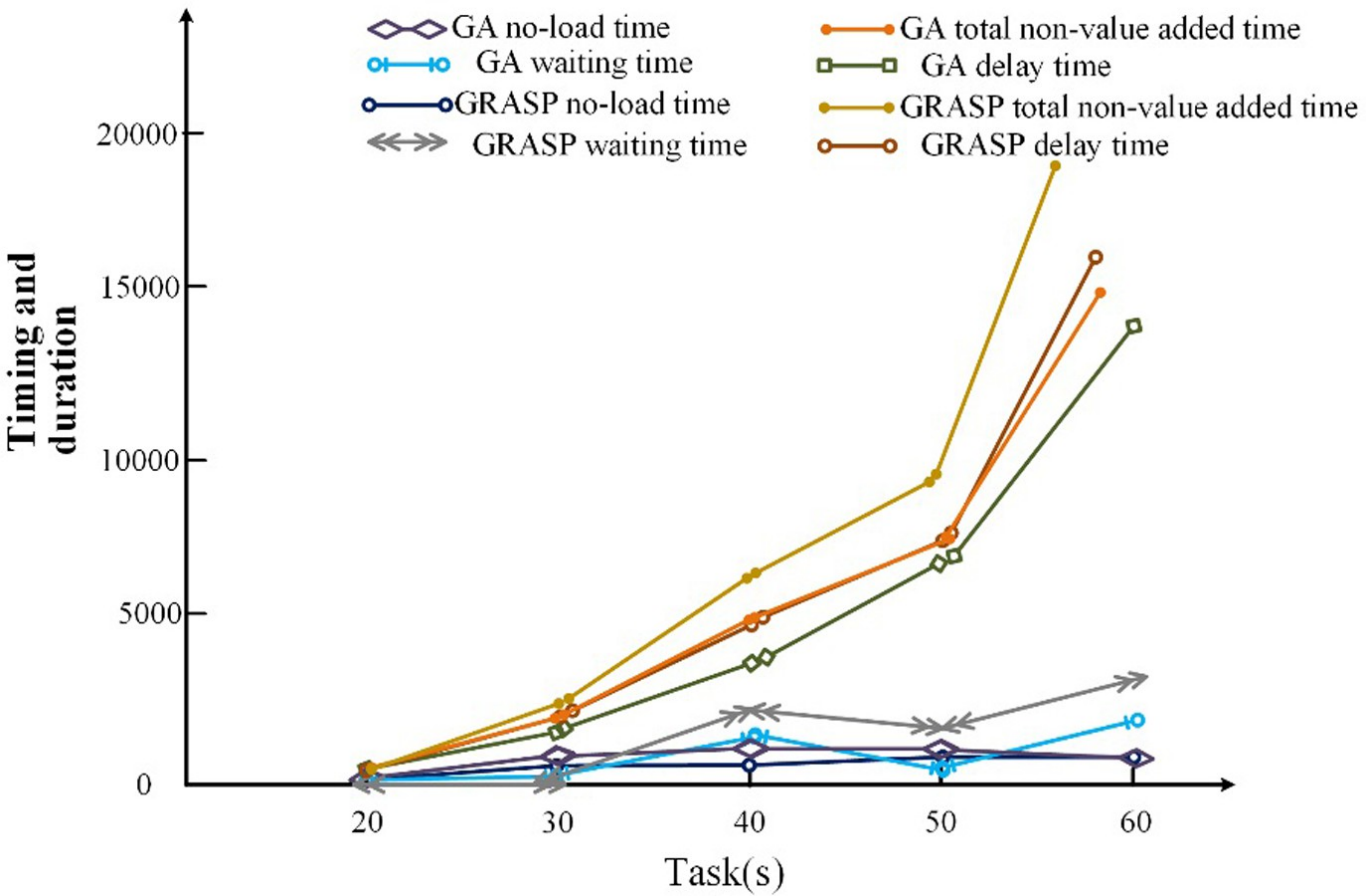
Comparison of scheduling results.

According to our findings, the simulated annealing algorithm’s total non-value-added time, waiting time, no-load time, and postponement time are higher than those of the GA algorithm. The detailed results can be seen in Fig 9.

### 5.5 Comparisons with not DT-based scheduling

**Fig 10:**
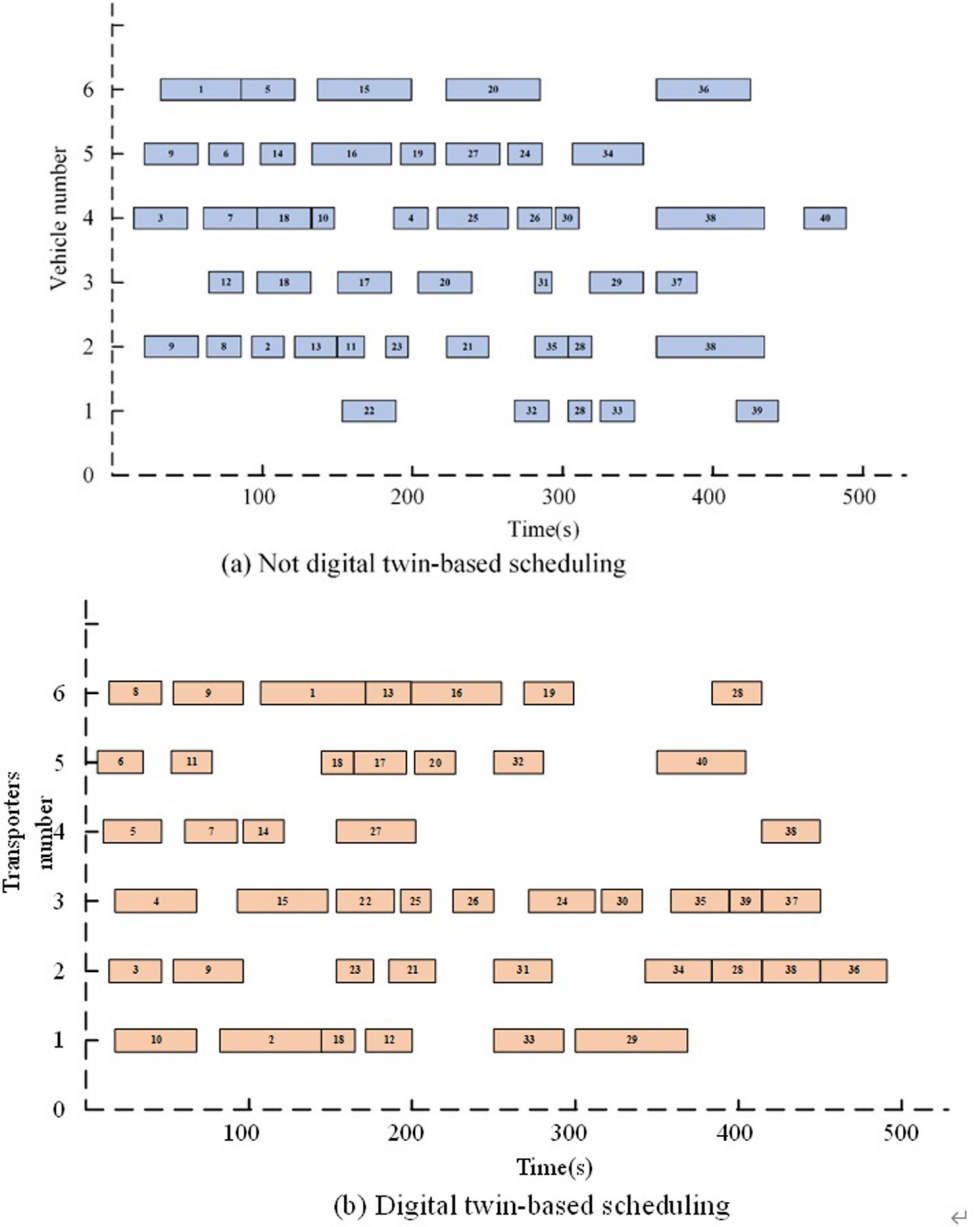
Gantt chart of the results of transporters and task blocks scheduling.

To showcase the advantages of employing a DT-based approach for dynamic scheduling of transporters, a shipyard multiple transporters scheduling system is developed, leveraging the unique strengths provided by this approach. To evaluate and compare the benefits and drawbacks of the two scheduling solutions, a comparison experiment is conducted using a practical block transportation task at shipyard. In Fig 10, the results of the transporters and task blocks assignment are presented in the form of a Gantt chart. The computing time and objective function values are summarized in Table 8.

**Table 8:** Optimization of objective function values and mean CPU time results.

Proposed Method	Objective Function Values	Mean CPU time
With DT	1432.2	250.629s
Not DT	1699.3	395.146s

During the scheduling and execution process, a malfunction was detected in transporter 9 by the job process status monitoring service at 261s, necessitating a three -minutes repair period. The multiple transporters scheduling system, leveraging the DT-based approach, employs predictive techniques to anticipate the consequences of the transporter malfunction on the execution plan. Additionally, the system validates whether the disrupted plan adheres to the existing constraints, ensuring that the revised schedule remains feasible and compliant with the requirements. In case it fails to meet the requirements, a re-scheduling will be initiated, involving reassignment of unfinished tasks and confirmation of the new plan in the twin space before replacing the current execution plan. Upon completion of re-scheduling, the final plan is obtained as depicted in Fig 10.

**Fig 11:**
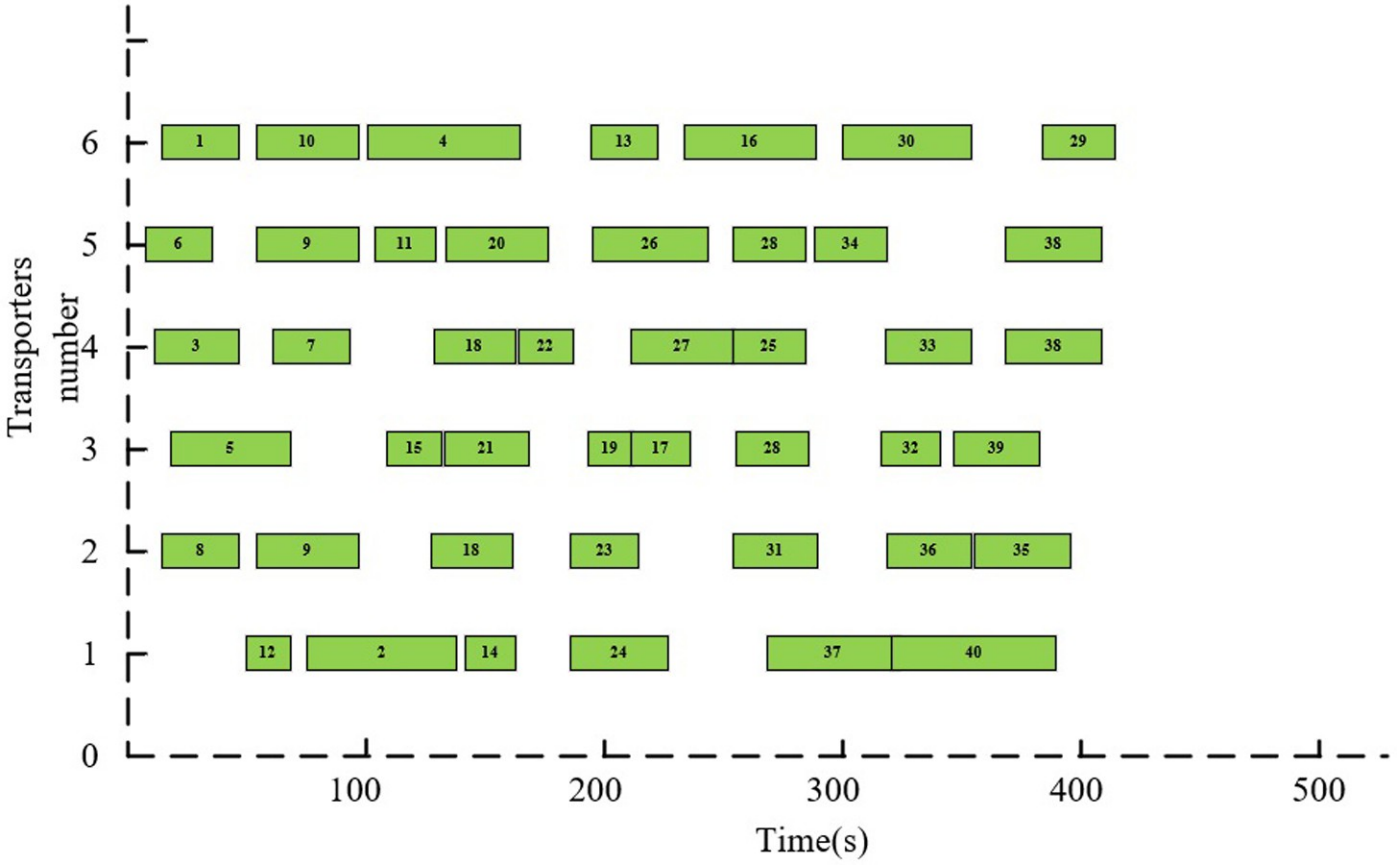
The ultimate DT-based scheduling solution.

As presented by Fig 11, the DT-based transporters scheduling method reschedules uncompleted block tasks when uncertainty in the scheduling process occurs, i.e., tasks on the faulty transporter are assigned to the earliest completed transporter. By analyzing the results of the DT system for transporter scheduling, the system can respond in a timely manner to uncertain events during yard scheduling operations while ensuring the optimality of its scheduling solutions.

Based on our analysis of the presented results, several observations can be deduced. When tackling small-scale instances, the CPLEX approach can optimally resolve them within a reasonable computational timeframe compared to GA. However, for large-scale mathematical scenarios, GA can yield improved solutions within an acceptable duration.

And the existence of the DT therefore makes it possible to interact the twin space with the physical space. The interaction between the two triggers timely rescheduling, which reduces the idle time of transporters, increases transport efficiency and thus reduces the maximum make span time. In other words, by the means of the closed-loop and iterative optimization between the service system and the twin space, the parameters in transporter scheduling become more and more precise and the scheduling optimality more and more optimal.

### 5.6 Sensitivity analyze

In order to verify the validity of the proposed methodology in this paper, a sensitivity analysis of the key parameters is performed in this section. However, since this paper has already investigated the comparison of results in different cases of various block numbers (*B* = {20,30,40,50,60}) and numbers of transporters (*T* = {4,5,6}) in subsection 4.2.

**Fig 12:**
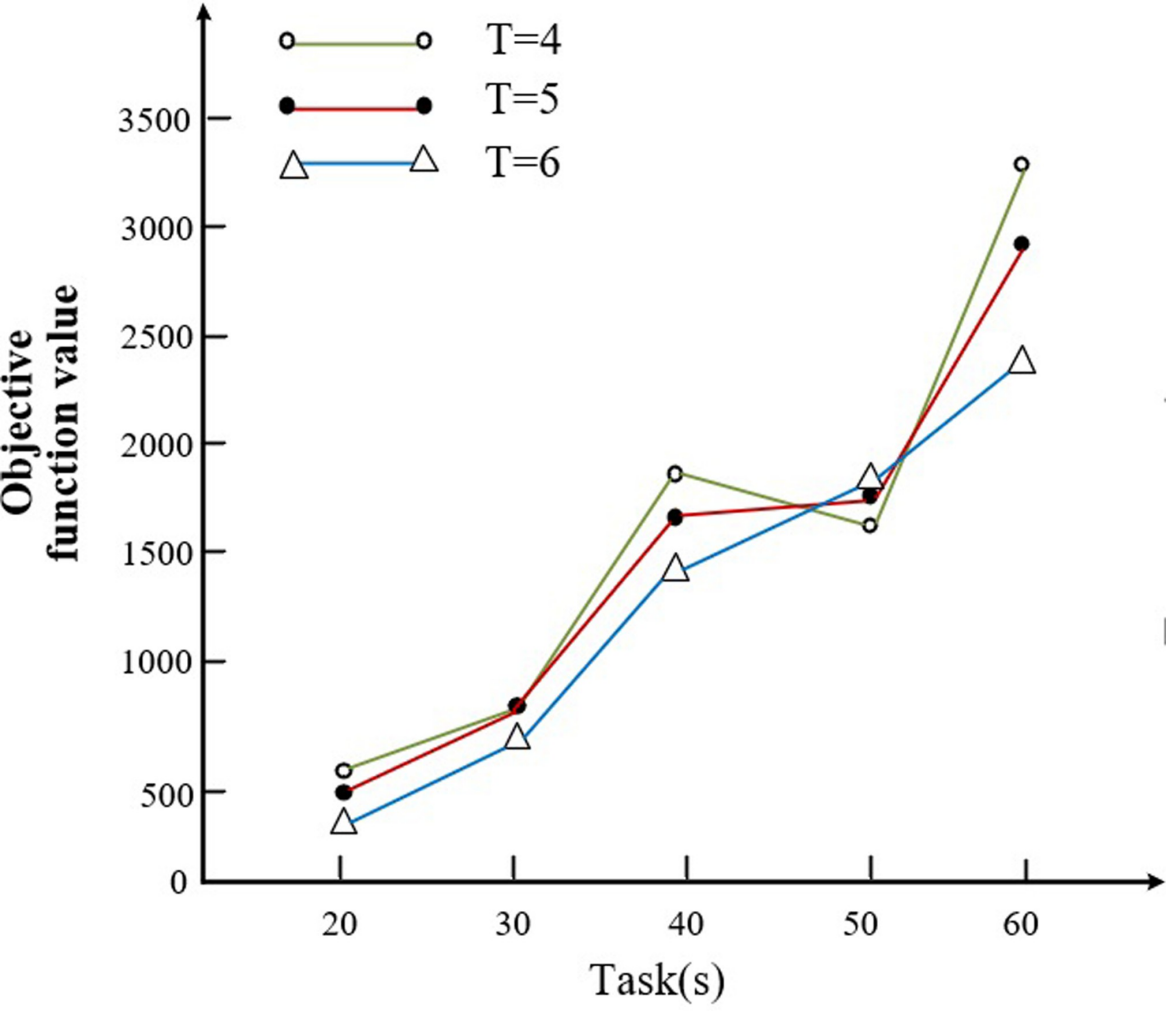
Comparison of scheduling results for different number of tasks and number of transporters.

Therefore, this subsection discusses the effect of the number of transporters on the objective function from the case of constant task size as well as the effect of the number of block tasks of different sizes on the objective function under the condition of fixed number of flatbed trucks. The details are shown in the following Fig 12.

**Fig 13:**
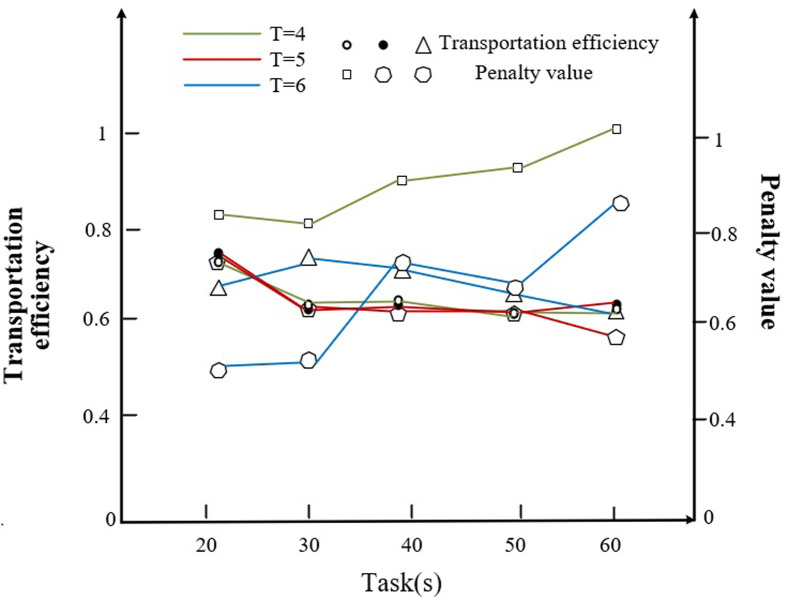
Corresponding transportation efficiency and penalty values for transporters for different numbers of tasks.

As we can see from Fig 12, with the increasing number of block tasks, the objective function value is relatively low when the number of transporters is 4, at which point the optimum is achieved. In addition, we go to analyze the transportation efficiency and the percentage of penalty value due to delay, delay and the number of transporters to the objective function for the cases of 4, 5 and 6 transporters, respectively, as shown in Fig 13.

From Fig 13 we can see that with the increasing number of tasks, when the number of transporters is 6, the transportation efficiency is higher, and its corresponding penalty value due to delay, delay, etc. is lower.

Through the sensitivity analysis above, we can check that the number of flatbed trucks and the task scale in different schemes have a strong impact on the objective function. Therefore, we cannot blindly increase the number of flatbed trucks in pursuit of transport efficiency in the actual operation process of the shipyard. This will instead lead to many other problems. Nor can we reduce the number of flatbed trucks in order to reduce investment, otherwise transportation efficiency cannot be guaranteed. Therefore, in summary, related shipyard management companies can refer to the solution method proposed in this question to make decision-making support when making choices.

## 6 Conclusions and future works

This study expands the application of DTs in the synchronization scheduling of multiple transporter transportation at shipyards. The scheduling process of transporters is vulnerable to uncertainties that can cause delays in task blocks and even interruptions in transportation. Given the inherent complexity and variability of transporter scheduling processes, it becomes crucial to promptly respond to disruptions and uncertainties that may arise during the synchronization transportation process. The emergence of DTs, however, presents a novel solution to this challenge. By reducing the total transportation time of transporters and enhancing transportation efficiency, the method has a significant impact on regular production and manufacturing at shipyards.

In order to solve the special problem, the optimization method of multiple transporters synchronization dynamic scheduling strategy based on DTs is proposed. Based on the presented method, we build a DT framework for transporter transportation and research that how the DT framework works. A mixed integer planning model is developed. The GA of dynamic scheduling based on DT is designed. Finally, the optimality and computational time of these solution methods are compared with different numbers of simultaneous constraints, DT-based scheduling, and not DT-based scheduling as examples. It is concluded that the GA and DT-based dynamic scheduling scheme is effective for the optimization of multiple transporters synchronization transportation scheduling at shipyards. The combination of the optimized algorithm for transporter scheduling and DT technology studied in this article is of great significance to digital shipbuilding. It will further enhance shipbuilding management capabilities and provide new insights for real-world transportation planning.

However, how to address the interference limitations of roads in the yard remains a limitation of this study. In actual transportation operations, some roads can only accommodate one transporter at a time. There are several key points for future research needs: (1) Whether there are constraints between block tasks should be considered. (2) Constructing high-fidelity virtual models and applying deep learning to a DT framework for synchronized truck scheduling of transporters to solve complex transporter scheduling problems with high dynamics and real-time performance. These will be the next research focus.

## Supporting information

S1 File (RAR)
